# Tunisian Maturity-Onset Diabetes of the Young: A Short Review and a New Molecular and Clinical Investigation

**DOI:** 10.3389/fendo.2021.684018

**Published:** 2021-07-29

**Authors:** Mariam Moalla, Wajdi Safi, Maab Babiker Mansour, Mohamed Hadj Kacem, Mona Mahfood, Mohamed Abid, Thouraya Kammoun, Mongia Hachicha, Mouna Mnif-Feki, Faten Hadj Kacem, Hassen Hadj Kacem

**Affiliations:** ^1^Laboratory of Molecular and Cellular Screening Processes, Center of Biotechnology of Sfax, University of Sfax, Sfax, Tunisia; ^2^Endocrinology Department, Hedi Chaker University Hospital, Sfax, Tunisia; ^3^Department of Applied Biology, College of Sciences, University of Sharjah, Sharjah, United Arab Emirates; ^4^Pediatric Department, Hedi Chaker University Hospital, Sfax, Tunisia

**Keywords:** MODY, genetic testing, Sanger sequencing, *GCK*, *HNF1A*, clinical exome sequencing

## Abstract

**Introduction/Aims:**

Maturity-Onset Diabetes of the Young (MODY) is a monogenic non-autoimmune diabetes with 14 different genetic forms. MODY-related mutations are rarely found in the Tunisian population. Here, we explored MODY related genes sequences among seventeen unrelated Tunisian probands qualifying the MODY clinical criteria.

**Materials and Methods:**

The *GCK* and *HNF1A* genes were systematically analyzed by direct sequencing in all probands. Then, clinical exome sequencing of 4,813 genes was performed on three unrelated patients. Among them, 130 genes have been reported to be involved in the regulation of glucose metabolism, β-cell development, differentiation and function. All identified variants were analyzed according to their frequencies in the GnomAD database and validated by direct sequencing.

**Results:**

We identified the previously reported *GCK* mutation (rs1085307455) in one patient. The clinical features of the MODY2 proband were similar to previous reports. In this study, we revealed rare and novel alterations in *GCK* (rs780806456) and *ABCC8* (rs201499958) genes with uncertain significance. We also found two likely benign alterations in *HNF1A* (rs1800574) and *KLF11* (rs35927125) genes with minor allele frequencies similar to those depicted in public databases. No pathogenic variants have been identified through clinical exome analysis.

**Conclusions:**

The most appropriate patients were selected, following a strict clinical screening approach, for genetic testing. However, the known MODY1-13 genes could not explain most of the Tunisian MODY cases, suggesting the involvement of unidentified genes in the majority of Tunisian affected families.

## Introduction

Maturity-Onset Diabetes of the Young (MODY) is a dominantly inherited form of non-autoimmune monogenic diabetes. Mutations in one of the key genes related to β-cell function and pancreas development are the main factors of this disease (1). So far, the Online Mendelian Inheritance in Man (OMIM) database has summarized 14 genes responsible for the emergence of MODY subtypes (OMIM # 606391) (2). More than 75% of MODY cases are the outcome of mutations in four genes [Hepatocyte Nuclear Factor (HNF) 4 Alpha (*HNF4A*), *HNF1A*, HNF 1 Beta (*HNF1B*), and glucokinase (*GCK*)] but their frequency varies among tested populations (3, 4). On the other hand, the development of clinical molecular diagnostic technologies has facilitated the discovery of new and unclassified genes related to MODY {Regulatory Factor X (*RFX6*) (5), NK6 homeobox 1 (*NKX6-1*) (6), and Insulin Receptor Substrate 1 (*IRS1*) (7) genes}. However, many young-onset diabetic cases exhibiting MODY-like phenotype remain genetically unexplained (MODY-X) (8, 9).

MODY gene mutations manifest clinically heterogeneous features in terms of age of onset, the pattern of hyperglycemia, and treatment modalities (9, 10). Therefore, an accurate molecular diagnosis of MODY is pivotal for early prognosis, treatment, and genetic counseling (1).

MODY represents less than 6% of entire diabetes cases (2, 11) and contributes to 1–6% of pediatric diabetes cases in European and North American populations (12). The factors such as undiagnosed cases, misdiagnosed patients as other types of diabetes (type 1 (T1D), type 2 (T2D), or gestational diabetes), and variable methods of clinical ascertainment (13, 14) result in inaccurate MODY prevalence reports globally (15).

**Figure 1:**
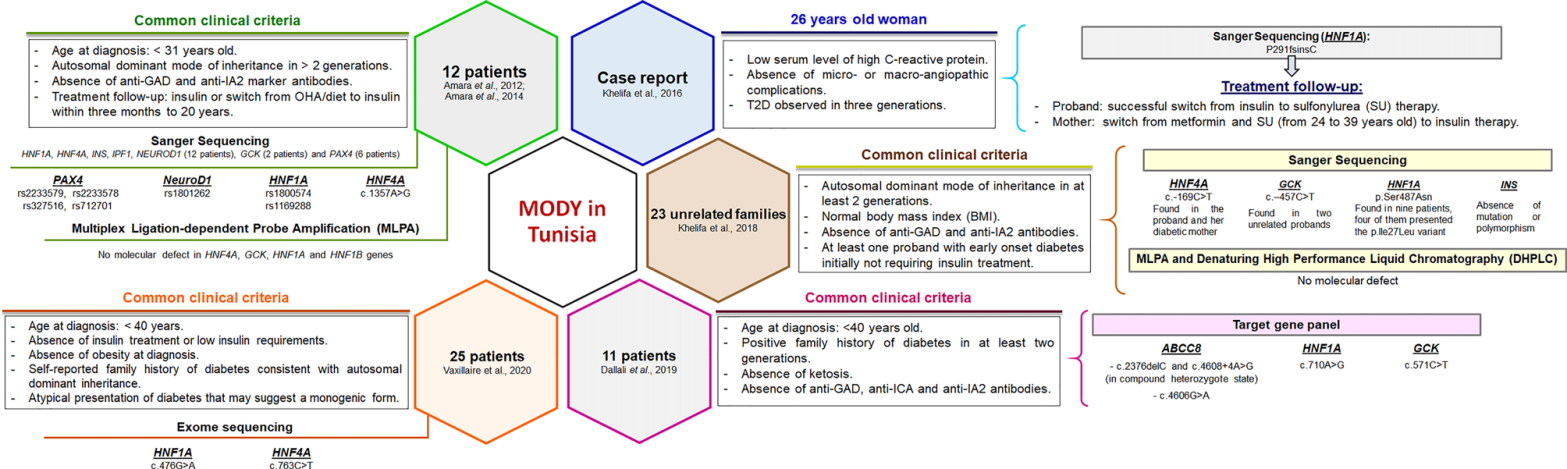
Clinical and genetic characterization of Tunisian MODY patients reported in previous studies. The clinical and familial inclusion criteria were specific for each study. Pathogenic variants and polymorphisms found in Tunisian patients were mainly reported in the most prevalent MODY genes. Functional studies have not been performed. OHA, oral hypoglycemic agents.

In Tunisia, a few MODY cohorts have been screened but the accurate prevalence is not known. The clinical and familial inclusion criteria were lucid but specific for each investigation (**Figure 1**). The first study identified c.1357A>G mutation in the *HNF4A* gene of one patient, whereas 11 patients were negative for mutations in *HNF4A*, *HNF1A*, *HNF1B*, *GCK*, Pancreatic and Duodenal Homeobox 1 (*PDX1*), insulin (*INS*), and Neuronal Differentiation 1 (*NEUROD1*) genes (16). Index cases of six out of 11 families were also negative for mutations in paired box 4 (*PAX4*) gene (17). The second study reported P291fsinsC mutation in the *HNF1A* gene (18). Khelifa et al. (19) also studied 23 unrelated families and screened four MODY genes (*INS*, *GCK*, *HNF1A*, and *HNF4A*) for mutations. Two variants were found in *GCK* (c.-457C>T) and *HNF4A* (c.-169C>T) genes of three unrelated probands. Dallali et al. (20) studied 11 suspected MODY patients and identified five heterozygous pathogenic variants in four patients {*GCK* p.Arg191Trp, *HNF1A* c.710A>G, adenosine triphosphate (ATP)-binding cassette, sub-family, member 8 (*ABCC8*) [(c.2376delC and c.4608+4A>G found at compound heterozygous state) and c.4606G>A]}. Another study has also reported two pathogenic variants in *HNF1A* (c.476G>A) and *HNF4A* (c.763C>T) genes (21).

During this study, a cohort of seventeen unrelated Tunisian probands was selected based on strict clinical and familial diagnostic criteria common for all MODY subtypes. These probands were screened and analyzed for genotype–phenotype correlations.

## Materials and Methods

### Participants and Clinical Data

Patients were recruited through the Departments of Endocrinology and Pediatric of Hedi Chaker University Hospital (Sfax, Tunisia). The study was approved by the Regional Committee of the Protection of Persons, Sfax, Tunisia (CPP SUD N°28/2019). Patients, their parents (if under the age of 18), and participating family members provided their written informed consent to participate in this study.

Details about the medical history of the patients and their extended family members (where available) were documented. Classification and diagnosis of diabetes were based on the latest American Diabetes Association guidelines (22). Diabetes diagnostic criteria were based on thresholds of fasting plasma glucose, and/or 2-hour (2-h) plasma glucose after a 75-gram (75-g) oral glucose tolerance test (OGTT), and/or glycated hemoglobin (HbA1c) measures. The clinical and metabolic data included: date of birth, gender, nationality, native place, age at diagnosis, diagnostic approach, body mass index (BMI), fasting plasma glucose, HbA1c, diabetes autoantibodies [islet cell antibody (ICA), anti-glutamic acid decarboxylase (GAD), anti-insulin and antibody to protein tyrosine phosphatase (IA-2)], C-peptide, serum insulin, lipid profile [total cholesterol, triglycerides, high-density lipoprotein (HDL) and low-density lipoprotein (LDL)], liver function profile [alanine aminotransferase (ALAT), aspartate aminotransferase (ASAT) and C-reactive protein (CRP)], kidney function profile (urine ketones, urea, creatinine, and uric acid), patient’s medical history and initial/current medications.

### Clinical Screening Strategy

Initially, the patients exhibiting (i) clinical symptoms of Maternally Inherited Diabetes Deafness syndrome (MIDD; OMIM # 520000), (ii) T1D confirmed by positive test for diabetes autoantibodies, or (iii) insulin resistance features (obesity, dyslipidemia, acanthosis nigricans, polycystic ovary syndrome…) were excluded.

The families included in this study fulfilled the following clinical MODY diagnostic criteria (1): a familial transmission of diabetes (two or more affected generations); (2) early-onset hyperglycemia (one or more members diagnosed under the age of 30); (3) absence of beta-cell antibodies; (4) personal history of unexplained beta-cell dysfunction (early repeated episodes of hypoglycemia or hyperglycemia); (5) non-insulin dependence (lack or low daily requirements of antidiabetic drugs for at least three years after the diagnosis); and (6) absence of obesity at diagnosis. Finally, a total of seventeen unrelated Tunisian pedigrees were recruited. Four families provided complete medical records with available family members for co-segregation analysis. The detailed clinical data of the probands from these four families are shown in **Table 1**.

**Table 1:** Clinical and molecular characteristics of MODY probands and their family members with diabetes.

Patient ID	Sex/Current age, years	Age at diagnosis, years	BMI^a^, kg/m^2^	Fasting plasma glucose^a^, mmol/L	HbA1c values (at diagnosis–last follow up), %	Diabeticketosis/ketoacidosis^a^	TreatmentFollow-up	Complications	Probability of MODY, %	Gene(RefSeq)	Variants	Genotype	dbSNP ID	MAF in GnomAD	Pathogenicity according to ACMG ^b^, SIFT^c^, Polyphen-2^d^ Provean^e,^ HSF^f^	References
F1-IV.2^†^	M/8.3	4.3	15.1	6.81	6.2–6.5	No	OHA for two years then shifted to diet.	No	75.5	*GCK* NM_000162.5	Exon 5: c.571C>T, p.Arg191Trp	CT	rs1085307455	7.96e^−06^	Pathogenic ^c,d^	ClinVar, VarSome, LitVar^‡^
F2-III.11	F/63	40	n.a.	n.a.	n.a.–9	No	Metformin	No	n.a.	*GCK* NM_000162.5	Exon 7: c.774C>T, p.(Gly258Gly)	CT	rs780806456	1.77e^−05^	Uncertain significance ^b^, affect splicing ^f^	Present study
										*KLF11* NM_003597.5	Exon 2 : c.185A>G, p.Gln62Arg	AG	rs35927125	9.13e^−02^	Benign ^c,d^	ClinVar, VarSome, LitVar^‡^
F2-IV.12^†^	M/33.3	27	17.35	29.5	13.2–12.2	ketoacidosis	Initial treatment on insulin (0.2 IU/kg/day). Shifted to OHA after two years. Current treatment with insulin at a dose of 0.3 IU/kg/day	No	24.4	*GCK* NM_000162.5	Exon 7: c.774C>T, p.(Gly258Gly)	TT	rs780806456	1.77e^−05^	Uncertain significance ^b^, affect splicing ^f^	Present study
										*KLF11* NM_003597.5	Exon 2 : c.185A>G, p.Gln62Arg	AG	rs35927125	9.13e^−02^	Benign ^c,d^	ClinVar, VarSome, LitVar^‡^
F2-IV.16	F/40	33	n.a.	n.a.	n.a.– 8	No	Metformin	No	n.a.	*GCK* NM_000162.5	Exon 7: c.774C>T, p.(Gly258Gly)	TT	rs780806456	1.77e^−05^	Uncertain significance ^b^, affect splicing ^f^	Present study
										*KLF11* NM_003597.5	Exon 2 : c.185A>G, p.Gln62Arg	GG	rs35927125	9.13e^−02^	Benign ^c,d^	ClinVar, VarSome, LitVar^‡^
F3-II.10	M/61	40	n.a.	n.a.	n.a.–8.5	No	Metformin and glimepiride	No	n.a.	*HNF1A* NM_000545.8	Exon 1 : c.293C>T, p.Ala98Val	CT	rs1800574	2.82e^−02^	Benign ^c,d^	ClinVar, VarSome, LitVar^‡^
F3-III.1^†^	M/30	22	25.59	8.5	8–9	No	Metformin and glimepiride	No	75.5							
F4-III.2	F/65	38	n.a.	n.a.	n.a.–9	No	Metformin	No	n.a.	*ABCC8* NM_001287174.2	Exon 25 : c.2978G>A, p.(Arg993His)	GA	rs201499958	3.59e^−05^	Tolerated ^c^, probably damaging ^d^, damaging ^e^	Present study
F4-IV.2	F/37	24	19	19.5	8.5–11	No	Diet for 3 years, then metformin for 2 years—before starting insulin (0.7 IU/kg/day) + metformin	Diabetic nephropathy and retinopathy	75.5			GG				
F4-IV.8^†^	M/33	19	19.5	23	10–13	Ketosis	Insulin (0.7 IU/kg/day) and metformin were started three years after the first diagnosis.		58			AA				

BMI, Body Mass Index; HbA1c, glycated hemoglobin; n.a., Not available; OHA, oral hypoglycemic agents. ^a^BMI and fasting plasma glucose values at first admission with/without the acute metabolic disorder. The probability of MODY was calculated only for the young-onset diabetes patients using MODY probability calculator. ^b,c,d,e,f^The prediction of the deleterious effect of variants using different tools. The estimated minor allele frequency for HNF1A p.A98V variant was around 3%; while the GCK p.(Gly258Gly) variant was absent in 50 healthy subjects. The restriction enzymes HaeIII and HinP1I were used to digest the PCR products covering the rs1800574 and rs780806456, respectively. ^†^Index case. ^‡^Variant found in LitVar (23).

A group of 181 people without clinical evidence of insulin resistance, abnormal glucose homeostasis, and/or autoimmune endocrine diseases served as a control in this study.

### Genetic Screening Strategy

Genomic DNA was extracted from the peripheral blood leukocytes using the phenol-chloroform-based protocol (24). The DNA assessment was performed through NanoDrop 2000 (ThermoFisher Scientific) and Qubit^®^ 2.0 fluorometer (Invitrogen, CA).

In the 17 unrelated participants, the entire exonic and consensus splice site regions of both *GCK* and *HNF1A* genes were screened for mutations by direct sequencing. Specific primers were designed using the Primer 3 program (25) and Refseq genes sequences. Primers and protocols of Polymerase Chain Reaction (PCR) are available on request.

Then, clinical exome enrichment was carried out using the TruSight One sequencing panel (Illumina, Inc. San Diego, CA, USA) targeting exonic regions of 4,813 genes associated with known clinical phenotypes. Only three patients with available family members’ medical records and DNA samples were investigated. All procedures were performed according to the manufacturer’s sample preparation protocol (Catalog No. FC-141-1007). Quantification and validation of the enriched libraries were performed using the Qubit dsDNA HS assay kit (Invitrogen) and 2100 Bioanalyzer Instruments (Agilent Technologies, Santa Clara, CA, USA). Generated libraries were paired-end sequenced (2 × 151 base pair) on a MiSeq instrument (Illumina) using the MiSeq Reagent kit V3 (300 cycles), according to the standard guidelines for preparing and loading samples on MiSeq.

### Bioinformatics Data Analysis

Default parameters from Illumina’s MiSeq Reporter (MSR) v2.6.2.3 software were used to analyze the data of Illumina’s clinical sequencing assay. After demultiplexing and FASTQ files generation, the Burrows–Wheeler Aligner (BWA) enrichment packages (BaseSpace, Illumina) were used to read and map the raw sequence data against the GRGh37 (hg19) human reference assembly, while the Genome Analysis Toolkit (GATK) software (MSR, BaseSpace, Illumina) was used for variant calling. The sequencing quality parameters obtained from the MiSeq sequencing platform were noted as 140 K/mm^2^ cluster density and 92.90% of clusters passing filter. In addition, 91.4% of bases showed a desirable quality score, at or above Q30. Data of all reads and variants were visually inspected through the Integrative Genomics Viewer tool (version 2.3) (26, 27), and almost all the genes had exons with enough coverage.

The bioinformatic analysis of the resulting variant call formats (VCFs) files was performed in the Illumina VariantStudio software (v3.0). This software provides access to specific tools and several resources including COSMIC (28), HGMD (29), ClinVar (30), and OMIM (31) that facilitate annotations at variant, gene, and transcript levels. The data of variants were gathered from different projects and databases including Single Nucleotide Polymorphism Database (dbSNP) (32), 1,000 genomes project (33), gnomAD database (V2.1.1) (34), and Exome Sequencing Project (ESP) (35). Several *in silico* prediction tools including SIFT (36), Polyphen-2 (37), and PROVEAN (38) were used to evaluate the effect of all missense variants reported in this study. Synonymous and splice site variants were evaluated *in silico* using splice site-altering scoring algorithm {Human Splice Finder (HSF) v3.1 (39)}. The Ensembl Variant Effect Predictor (VEP) (40) and VarSome database (41) were also used to access multiple databases, prediction tools, and publications at a single website. The guidelines of the American College of Medical Genetics and Genomics and the Association of Molecular Pathologists (ACMG/AMP) were applied to the newly identified variants (42).

### Filtering Strategy

Initially, heterozygous variants identified by Sanger sequencing were analyzed according to their impact’s severity (missense, nonsense, splice sites, insertion–deletion (indel), in frame and frameshift) and their frequencies [minor allele frequency (MAF) below 1%] in multiple public databases. Two additional parameters were considered in our study in order to analyze the totality of MODY genes, including (i) the uncommon synonymous variants predicted to affect the mRNA splicing but not previously described in the HGMD Professional, and (ii) the autosomal recessive mode of inheritance (homozygous and compound heterozygous variants).

Subsequently, we established a list of 133 genes previously known to be involved in MODY transmission (MODY1-13 genetic subtypes), and/or monogenic (isolated or syndromic) diabetic forms or associated with T2D and glucose metabolism (**Table S1**). We particularly focused on variants previously associated with T2D or detected in the 133 genes. Finally, the retained variants within all enriched genes were analyzed according to their impact’s severity and frequencies (MAF below 1%) in GnomAD.

### Sanger Sequencing

Sanger sequencing of available family members was carried out to confirm the familial co-segregation of retained variants. Primer sequences and amplification protocols are available on request. PCR products were purified using the PureLink Quick Gel Extraction Kit (Thermo Fisher Scientific) and then sequenced on an ABI 3100-4 automated sequencer (Applied Biosystems Inc, Foster City, CA, USA) according to the manufacturer’s instructions. Data of Sanger sequencing were analyzed using Bioedit software v7.1.3.

## Results and Discussion

Our genomic analysis included seventeen clinically diagnosed MODY patients. Their age of diagnosis ranged from 4 to 30 years. Most patients had a normal BMI at diagnosis; except one proband found to be overweight (**Table 1**). Blood glucose control was not ideal for some patients as the median HbA1c was 9.7% (6.2–13.2%). Diabetes treatment at diagnosis was heterogeneous. Two participants (2/17; 12%) were controlled with diet, fourteen (14/17; 82%) were treated with oral hypoglycemic agents (OHA) and 6% (1/17) with insulin. During the follow-up, we noticed that the treatment of only one patient, among the 14 participants previously treated with OHA, switched to diet. Additionally, we changed the treatment of the two patients initially controlled with diet into a combination of insulin and oral agents (**Table 1**). The long-term micro and macro-vascular complications were rarely found (11% of the probands).

The patients’ collected data were used to calculate the probability of MODY based on an online formula available at www.diabetesgenes.org (8). It is noteworthy that the probability of MODY occurrence increases among negative pancreatic autoantibodies patients. The mean probability of MODY was greater than 62% in our patients with young-onset diabetes (**Table 1**).

### Screening of *GCK* Gene and Clinical Phenotype Analysis

We screened the promoter region, each of the ten exons and their flanking intron sequences specific for the pancreatic isoform of the *GCK* gene (NM_000162.5) in seventeen unrelated index cases. We identified two genetic variants (pathogenic and of uncertain significance) in two unrelated index cases. **Table 1** shows the clinical, laboratory and genetic characteristics of patients with *GCK* gene variants.

**Figure 2:**
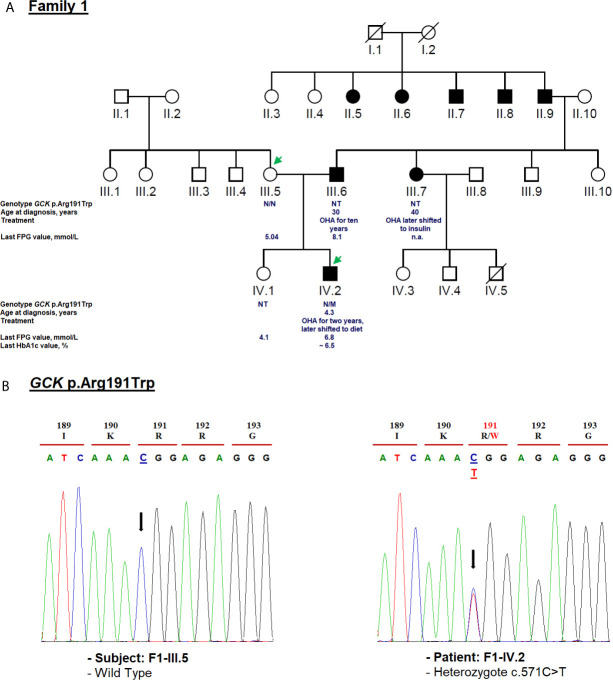
Mutation analysis of the *GCK* gene. Panel **(A)** Pedigree of the family F1 identified with heterozygous *GCK* variant (NM_000162.5: c.571C>T, p.Arg191Trp). The generations within the family are indicated by roman numerals. Squares and circles represent male and female family members, respectively. Normal individuals are shown as a clear symbol. Black-filled symbols denote patients with diabetes. A line through a symbol denotes the deceased. Green arrows indicate available DNA members. OHA, oral hypoglycemic agents; FPG, fasting plasma glucose; NT, not tested. The genotype is shown underneath each symbol. M and N denote mutant and wild-type alleles, respectively. The age of diabetes onset, glycemic control (the latest FPG or HbA1c measurements), and treatment follow-up are indicated directly below the genotype. Panel **(B)** Electropherogram analysis of *GCK* gene in the family F1. Mutated nucleotide on the chromatographs is depicted with an arrow. Amino acid substitution is indicated in red.

The first substitution was the c.571C>T mutation (exon 5), and leads to the replacement of arginine to tryptophan in codon 191 (p.Arg191Trp; rs1085307455; **Figure 2B**). This mutation was observed in gnomAD with a MAF of 7.96e^−06^, but it remained absent in the large population cohorts (NHLBI-ESP and the 1000 Genomes Project). Bioinformatics analysis predicted that the *GCK* p.Arg191Trp mutation could affect the protein structure and function (43). Other computational studies have shown that the Arg191 residue contributes to the transformation of the enzyme’s spatial conformation (43, 44). Nevertheless, *in vivo* analysis have revealed a decreased thermostability of p.Arg191Trp mutant protein that ultimately leads to full kinetic inactivation of glucokinase (45).

The 191Trp residue was primarily found in Caucasian gestational diabetic subjects who did not meet MODY diagnostic criteria (46). Indeed, the c.571C>T mutation has been previously identified in individuals (pregnant women, children and adolescent) with MODY2 phenotype of British (46), Italian (47), Korean (48), Belgian (49), Norwegian (50), French (51), Japanese (52), Brazilian (53), Chinese (45) and Tunisian (20) descent. HGMD Professional has also reported three other amino acid substitutions on the same residue (p.Arg191Leu/Gln/Glu) related to MODY2 (47, 54, 55).

We identified c.571C>T mutation (p.Arg191Trp) in the F1-IV.2 proband. This proband was referred to the pediatric endocrinology clinic for mild fasting hyperglycemia of 6.81 mmol/L. The fasting blood glucose test was done as the proband exhibited persistent diabetes-specific symptoms (polyuria and polydipsia) at the age of 4.3. At admission, his weight, height and BMI were recorded as 17 kg (−0.5 SDS), 106 cm (−1 SDS), and 15.1 kg/m^2^, respectively. His HbA1c was mildly elevated (6.2%) (**Table 1**) whereas the value of fasting C-peptide level was lower (0.07 pmol/ml) than the reference range (RR: 0.37–1.47). A slight increase in the glucose level (5.76 to 8.78 mmol/L) during a 2-h OGTT confirmed the impaired glucose tolerance diagnosis. The biochemical and metabolic parameters were found to be within the physiological limits without clinical characteristics of insulin resistance. The strong paternal family history of T2D is displayed in **Figure 2A**. The proband’s father (F1-III.6) was diagnosed with T2D at the age of 30. His fasting blood glucose measurements have always been below 8.5 mmol/L and he is currently on OHA therapy. The proband’s paternal aunt (F1-III.7) was diagnosed with T2D at the age of 40 and started OHA as well but she is currently on insulin therapy. However, cascade genetic testing confirmed the absence of this mutation in the proband’s mother (F1-III.5; **Figure 2B**). The proband’s father refused biochemical assessment, genetic testing or counseling. Genetic testing was useful in this case as it improves the clinical course and guided the management of our proband. The OHA therapy of this boy was immediately ceased (**Table 1**). Lifestyle counselling was provided for healthy eating and physical activity that stabilized his health. He was examined every 6 months at the outpatient clinic, and his HbA1c always remained stable at 6.5% (**Table 1**).

The clinical phenotype of our MODY2 subject (F1-IV.2) was similar to that described in previous reports (56). We recommended fasting blood glucose measurement, 2-h OGTT, and genetic testing of this mutation for each at-risk or affected family members. Hyperglycemia in F1-III.6 and F1-III.7 probands must be pharmacologically managed according to their genotypes. Furthermore, the c.571C>T mutation has already been reported in another unrelated Tunisian pedigree (20). The main reason for this mutation has never been explained that whether it is due to the gene founder effect or mutational hotspot in Tunisia. Indeed, the *GCK* small domain has been specifically considered a mutation hotspot of Southern Italy (57). Of note, it has been observed that some subjects suffering from *GCK*-MODY were treated with low daily doses of OHA and/or insulin (50, 56). Therefore, the identification of this mutation among a large cohort of Tunisian patients with mild non-progressive early-onset hyperglycemia or gestational diabetes can facilitate early diagnosis of this disease and allow for a personalized treatment. It is noteworthy that according to previous studies, the treatment for *GCK*-MODY outside of pregnancy is not required (58, 59).

**Figure 3:**
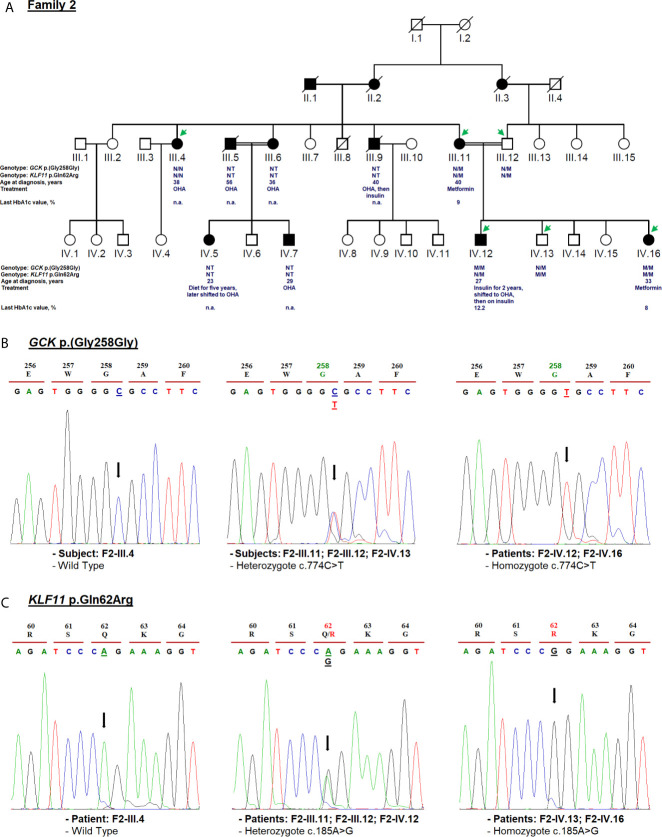
Mutation analysis of the *GCK* and *KLF11* genes. Panel **(A)** Pedigree of the family F2 identified with *GCK* [NM_000162.5: c.774C>T, p.(Gly258Gly)] and *KLF11* variants (NM_003597.5: c.185A>G, p.Gln62Arg). OHA, oral hypoglycemic agents; NT, not tested; n.a, Not available. M and N denote mutant and wild-type alleles, respectively. Clinical information such as the age of diabetes onset, treatment follow-up, and last HbA1c measurement is indicated directly below the genotype for p.(Gly258Gly) and p.Gln62Arg variants. Panel **(B)** Electropherogram analysis of *GCK* gene in the family F2. Panel **(C)** Validation of the *KLF11* c.185A>G variant segregation by Sanger sequencing. Mutated nucleotides on the chromatographs are depicted with an arrow. Amino acid substitution is indicated in red.

We also found, in the F2-IV.12 proband, a novel c.774C>T variant at exon 7 of the same gene resulting in an exonic silent substitution [p.(Gly258Gly); rs780806456; **Figure 3B**]. This variant has been reported in gnomAD (MAF = 1.77e^−05^), but not observed in healthy control subjects (n = 50). It was classified according to ACMG/AMP as a variant of uncertain significance (**Table 1**). Indeed, no data are available about its co-inheritance or association with any medical condition (mainly disorders of glucose metabolism).

The F2-IV.12 proband came to the emergency service, at 27-year-old, suffering from acute hyperglycemia (29.5 mmol/L). During clinical examination, his height, weight, BMI and blood pressure were recorded as 178 cm, 55 kg, 17.35 kg/m^2^, and 110/70 mmHg, respectively. Initial HbA1c was elevated to 13.2% with an acute metabolic complication of diabetes (ketoacidosis). Mild osmotic symptoms (polyuria, polydipsia) were noticed at the age of 16 but there was no medical history of permanent neonatal diabetes. The uncontrolled hyperglycemia and recurrent infections might have caused the ketoacidosis condition. Biochemical analysis revealed 1.02 mmol/L of triglycerides (RR: 1.5–2.8), 1.84 mmol/L of total cholesterol (RR: 4–6), 0.92 mmol/L of HDL, 0.46 mmol/L of LDL, 55 U/L of ASAT (RR: 10–40), 31 U/L (RR: 10–50) of ALAT, 81.80 mg/L of CRP (RR: <6), 384 µmol/L of creatinine (RR: 80–115), 581 µmol/L of uric acid (RR: 220–420), and 10.76 mg/day of microalbuminuria. The thyroid function tests were normal. Ultrasonography revealed the normal shape of kidneys, pancreas, and liver. Initially, continuous insulin therapy was prescribed, to normalize the blood glucose, followed by a gradual dose reduction. The HbA1c of this proband decreased to 5.2% at the 6-month follow-up and remained stable for 24 months. His renal function was also stable during this period. Biochemical analysis revealed triglycerides of 0.44 mmol/L, total cholesterol of 2.62 mmol/L, HDL of 1.22 mmol/L, LDL of 1.2 mmol/L, and creatinine of 67.47 µmol/L. After examining these results, the treatment of proband was switched from very low-dose insulin (0.2 units (IU)/kg/day) to OHA for a few months. Indeed, the fasting blood glucose control was not satisfactory as the HbA1c values were noted above 9%. Currently, the F2-IV.12 proband receives insulin at a dose of 0.3 IU/Kg/day (**Table 1**). During the last medical checkup, his HbA1c was noted as 12.2% because he failed to properly follow the hypoglycemic regimen. Diabetic retinopathy or retinitis pigmentosa was not observed during the fundus examination. Three siblings of the mother (F2-III.4, F2-III.6, F2-III.9) and their offspring (F2-IV.5, F2-IV.7) suffered from T2D as presented in **Figure 3A**. All the affected family members are on OHA therapy since the diagnosis of T2D and there are no micro- and macro-vascular diabetic complications in all affected proband’s family.

Cascade genetic testing revealed rs780806456 in F2-IV.12 and F2-IV.16 patients in the homozygous state (**Figure 3B**). The co-segregation analysis of rs780806456 showed that there is no correlation with diabetes in affected family members whose DNA was available for genetic testing (**Figure 3B**). Indeed, this variant was inherited from consanguineous parents (F2-III.11 and F2-III.12) who were heterozygous carriers and was absent in the maternal aunt of the proband (F2-III.4). Therefore, we could not confirm whether this variant is responsible for MODY.

The c.774C>T variant was predicted *in silico* to affect the mRNA splicing process through the creation of a new cryptic donor splice site or an exonic splicing silencer (ESS) site. Therefore, further functional investigation is required to evaluate its role in the phenotype of carrier patients. A recent *in vitro* study has supported the pathogenic effect of novel or rare synonymous *GCK* gene variants on splicing (60).

The clinical presentation of the F2-IV.12 proband did not meet classic MODY2 diagnostic criteria. Generally, patients suffering from *GCK*-MODY are asymptomatic with mild non-progressive hyperglycemia that is incidentally diagnosed (4). Their slightly higher HbA1c levels typically range from 5.6 to 7.3% at ≤40 years, and 5.9 to 7.6% at >40 years (61). *GCK*-MODY patients are usually not prone to long-term micro- or macro-vascular diabetic complications and are well responsive to diet without any pharmacological intervention (9, 62).

### Screening of *HNF1A* Gene and Clinical Phenotype Analysis

*HNF1A* gene mutation analysis was also conducted in all index cases. The clinical, laboratory, and genetic characteristics of the patients with *HNF1A* relevant variants, are summarized in **Table 1**.

**Figure 4:**
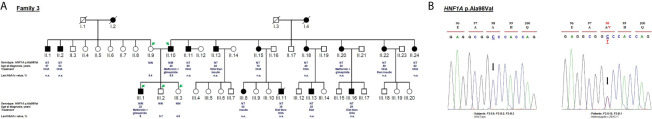
Mutation analysis of the *HNF1A* gene. Panel **(A)** Pedigree of the family F3 identified with *HNF1A* variant (NM_000545.8: c.293C>T, p.Ala98Val). OHA, oral hypoglycemic agents; NT, not tested; n.a, Not available. M and N denote mutant and wild-type alleles, respectively. The age of diabetes onset, treatment follow-up, and last HbA1c measurement are indicated directly below the genotype (were available). Panel **(B)** Electropherograms showing co-segregation of the heterozygous variant c.293C>T in *HNF1A* gene to the phenotype observed in the proband and his father. Proband III.1 and his father II.10 carry heterozygous alleles (C/T) linked to the phenotype whereas non-affected subjects (mother II.9 and siblings III.2, III.3) carry homozygous alleles (C/C). Mutated nucleotide on the chromatographs is depicted with an arrow. Amino acid substitution is indicated in red.

The heterozygous NM_000545.8: c.293C>T variant causes missense change in the protein from alanine to valine in residue 98 (p.Ala98Val; rs1800574). This variant co-segregates with diabetes in affected members (F3-II.10 and F3-III.1; **Figure 4B**). The c.293C>T variant is localized in the DNA-binding domain. It was first reported to be associated with a decrease in serum C-peptide and insulin responses to an oral glucose challenge (63). Then, several reports have demonstrated its mild impact on HNF1α function (64). The *in silico* analysis based on SIFT (score = 0.09) and Polyphen-2 (score = 0.229) predicted that this substitution is benign. We considered p.Ala98Val as a non-pathogenic variant due to its high frequency in the general population (MAF = 0.0282 according to GnomAD) and Tunisian control group (n = 181 subjects; MAF = 0.03; **Table 1**). rs1800574 frequency in diabetic patients and control groups has been widely studied in different Asian and European populations (65–67). Locke et al. (68) have reported that p.Ala98Val is not associated with the age during the diabetes diagnosis of *HNF1A*-MODY individuals. Previous studies have reported that there is no relationship between this variant and gestational diabetes in Danish (69), Polish (70), and Turkish (71) women. The role of *HNF1A* p.Ala98Val variant in early and late-onset of diabetes and MODY 3 differs between populations (67, 72, 73).

The c.293C>T variant was identified in a 22-year-old man (F3-III.1) with a 12-month history of polydipsia, polyuria and had lost 6 kg weight. At admission, the patient’s BMI was recorded as 25.59 kg/m^2^ (82 kg weight and 179 cm height) and physical examination depicted normal vital signs. Laboratory investigations revealed 8.5 mmol/L fasting blood glucose and 8% HbA1c value (**Table 1**) with no acute metabolic disorder (no ketosis). Biochemical analysis showed normal levels of triglycerides, total cholesterol, HDL, LDL, ASAT, ALAT, CRP, and creatinine. He received OHA (metformin and glimepiride) for eight years. During the last medical checkups, his plasma glucose level was not satisfactory (HbA1c around 9%) revealing that he was not adhering to the hypoglycemic regimen. Diabetic retinopathy or retinitis pigmentosa were not noticed in fundus examination. The strong paternal family history of T2D is displayed in **Figure 4A**. All affected family members had been on diet control followed by, or not, OHA therapy since the diagnosis of T2D. Micro- and macro-vascular diabetic complications were not observed in the affected proband’s family. They have no clinical evidence of insulin resistance syndrome, cardiovascular or renal disorders. The proband’s father (F3-II.10) was diagnosed with diabetes at the age of 40 and treated with OHA for 21 years (**Table 1**). The proband’s mother (F3-II.9) and siblings (F3-III.2; F3-III.3) had normal blood glucose responses two hours after the administration of 75-g of glucose solution, and normal HbA1c levels (**Figure 4A**). The proband’s siblings were negative for all diabetes autoantibodies. The MODY phenotype was suspected for this family based on the clinical features such as the early onset of diabetes, and management of diabetes with long-term OHA therapy.

Clinical expression of MODY3 considerably varies depending upon the genetic predisposition (*HNF1A* gene SNPs) and environmental factors (physical activity and diet) (74, 75). Therefore, some MODY3 patients may not meet all classical diagnostic criteria. Most of the patients are very sensitive to low doses of sulfonylureas (4) which is usually effective in children and young adults for decades, until the production of beta-cell insulin is severely decreased (64). MODY3 patients are prone to cardiovascular and microvascular diabetes complications (64, 76). The F3 family did not fulfill the classical diagnostic criteria of MODY3, and therefore it cannot be concluded that the p.Ala98Val variant is responsible for the described clinical phenotype.

### Clinical Exome Sequencing and Variants Detection

By screening *GCK* and *HNF1A* genes, only one family (family F1) was classified as MODY2. Subsequently, clinical exome analysis was performed on three unrelated index cases (F2-IV.12, F3-III.1, F4-IV.8) selected based on the available family members’ medical records and DNA samples. Therefore, two genetic variants have been identified in two unrelated patients.

The known variant c.185A>G (p.Gln62Arg; rs35927125) was detected in the Kruppel-like factor 11 (*KLF11*) gene (NM_003597.5). This variant was identified in the F2-IV.12 index case, his affected mother, normoglycemic father (with heterozygous genotype AG), affected sister, and normoglycemic brother (with homozygous genotype GG) (**Figure 3C**). rs35927125 was already present in public SNP databases as a common missense variant with a 9.13e^−02^ frequency of the G allele according to gnomAD. It was predicted as benign by multiple bioinformatics tools. However, Neve et al. (77) showed that the p.Gln62Arg variant impairs the activation of insulin promoter and reduces insulin expression levels in pancreatic beta cells. They also demonstrated a decrease in plasma insulin after an oral glucose challenge in the subjects carrying the 62Arg allele. rs35927125 was found to be significantly associated with T2D in North European populations (77). However, several association studies have reported contrasting results. Three studies of 8,676 Caucasian Northern-European individuals (78), 1,818 Japanese participants (79), and 1,337 Pima Indians (80) including case-control and/or family-based samples failed to replicate the association between *KLF11* p.Gln62Arg with T2D or other insulin-related quantitative traits (78). Previous studies disputed the *KLF11* gene association with MODY making the genetic diagnosis more challenging (12, 81).

For F4-IV.8 proband, clinical exome sequencing allowed the identification of a novel homozygous missense variant (c.2978G>A) in *ABCC8* gene (NM_001287174.2) at exon 25. This substitution leads to the replacement of arginine to histidine [p.(Arg993His); rs201499958; **Table 1**]. This variant has already been reported only in the gnomAD global population dataset (MAF = 3.59e^−05^), but its co-inheritance or association with any pathological condition is not confirmed. It was classified according to ACMG/AMP as of uncertain significance. Its pathogenicity was predicted as tolerated (score = 0.065), probably damaging (score = 0.945) and damaging (score = −2.57) by SIFT, Polyphen-2 and PROVEAN, respectively. Arg993 residue is a highly conserved amino acid, located in the flexible linker of SUR1 protein that follow the first nucleotide binding domain (NBD1) (82).

**Figure 5:**
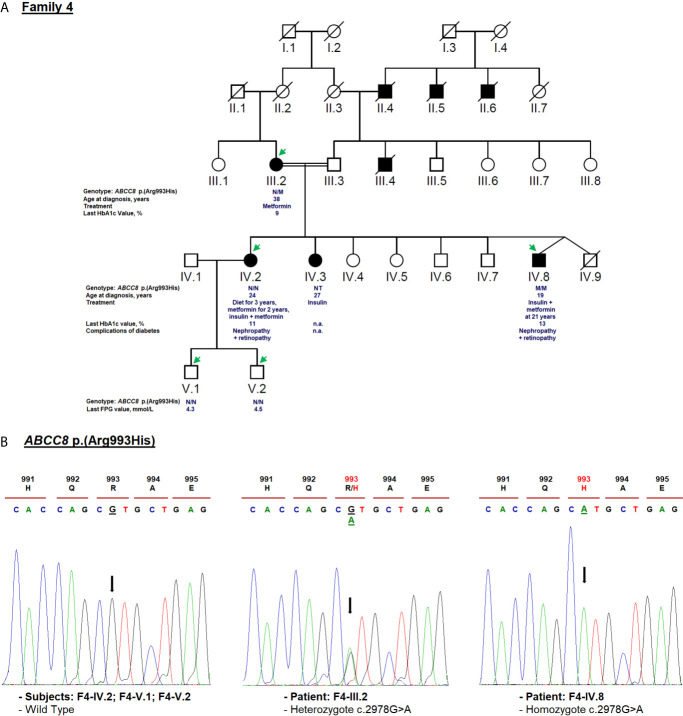
Mutation analysis of the ABCC8 gene. Panel **(A)** Pedigree of the family F4 identified with ABCC8 variant [NM_001287174.2: c.2978G>A, p.(Arg993His)]. FPG, fasting plasma glucose; NT, not tested; n.a, Not available. M and N denote mutant and wild-type alleles, respectively. The age of diabetes onset, glycemic control (the latest FPG or HbA1c measurements), complications of diabetes, and treatment follow-up are indicated directly below the genotype. Panel **(B)** Validation of the ABCC8 c.2978G>A variant segregation by Sanger sequencing. Mutated nucleotide on the chromatographs is depicted with an arrow. Amino acid substitution is indicated in red.

Diabetes symptoms were not observed in the F4-IV.8 index case when a fasting plasma glucose test was incidentally performed at the age of 19 in another medical establishment. He was tested because his mother (F4-III.2) and sisters (F4-IV.2; F4-IV.3) were diagnosed with diabetes and his blood glucose level was noted as 19.5 mmol/L. The laboratory findings of his initial diagnosis were not available. He was prescribed insulin treatment but he did not use it regularly. At the age of 21, he visited an emergency service for ketosis. At that time, his fasting blood glucose level and HbA1c were noted as 23 mmol/L and 10%, respectively (**Table 1**). Physical examination depicted normal vital signs with a BMI of 19.5 kg/m^2^. He was treated with insulin (0.7 IU/kg/day) and metformin. The proband refused to use the insulin, that worsened the glycemic control and led to early diabetic microvascular complications (diabetic nephropathy and retinopathy) at the age of 25. During the last medical checkup, an increased in HbA1c (13%) was noted because of irregular insulin usage. The laboratory examination also showed abnormal hepatic and renal functions. This patient was not followed up after the age of 30. His mother (F4-III.2) was diagnosed with T2D at the age of 38. Metformin was prescribed to manage plasma glucose levels and an HbA1c of 9% was noted at the last follow-up (**Table 1**). The elder sister (F4-IV.2) was diagnosed with T2D at the age of 24 and her BMI was 19 kg/m^2^ (**Table 1**). She is currently being treated with insulin (0.7 IU/kg/day) and metformin and her last follow-up HbA1c was 11% (**Table 1**). She suffered from diabetic nephropathy and retinopathy at the age of 32. His 35 years old sister (F4-IV.3) was diagnosed with gestational diabetes at the age of 27 and insulin therapy was recommended (**Figure 5A**). Unfortunately, her laboratory and clinical follow-up were not available. Interestingly, diabetes autoantibodies were negative for both sisters of the proband.

Cascade genetic screening identified the same mutation in the proband’s mother in heterozygote state, but the sister (F4-IV.2) and her children (F4-V.1, F4-V.2) were negative (**Figure 5B**). The *ABCC8* p.(Arg993His) missense variant was either *de novo* or inherited from the father. Unfortunately, proband’s father (F4-III.3) and affected sister (F4-IV.3) were not available for genetic testing. Further investigation needs to be conducted about the role of *ABCC8* p.(Arg993His) variant in hyperglycemia of this family. It has been reported that heterozygous carriers of *ABCC8* p.L171F mutation developed adult-onset diabetes, while homozygous carrier developed hyperinsulinemic hypoglycemia at neonatal period and diabetes later in life (83, 84).

This study revealed the absence of previously described or novel pathogenic variants within all enriched genes. This study can be further expanded in the future by (i) recruiting more MODY families to estimate MODY2 prevalence in Tunisia, (ii) conducting functional characterization of variants of uncertain significance to validate their effects on hyperglycemia, and (3) complete testing of other affected/unaffected family members to track variants co-segregation in each family.

The clinical inclusion criteria for patients in this study were rigorous compared to other Tunisian studies (**Figure 1**). In recent years, the recruitment criteria of MODY patients have been modified than originally defined by Tattersall and Fajans (85). Non-traditional MODY characteristics including the age of onset, obesity, the presence of diabetes autoantibodies and ketonuria, and treatment modalities have been evaluated (10). This study included patients with decompensated diabetes at study inclusion. Indeed, the absence of ketonuria was believed to be a definite criterion to differentiate T1D from MODY patients. However, the presence of ketonuria has been reported among patients with *HNF1A* (86, 87), *PDX1* (88), *NEUROD1* (89), and *INS* (90) mutations. The patients involved in this study presented a less severe metabolic profile (BMI <26 kg/m^2^). But, we exclude unintentionally carriers of pathogenic/likely pathogenic variants within the most common MODY genes (*HNF4A* and *HNF1A*) (91, 92). Recently, Vaxillaire et al. (21) analyzed a cohort of Mediterranean patients suspected with monogenic diabetes and observed that carriers of pathogenic variants had the lowest BMI.

This is not the first study in Tunisia that describes the genetic etiology of MODY/monogenic diabetes in patients with early-onset diabetes. Overall, 89 unrelated MODY suspected Tunisian participants (including this study) have been investigated using targeted gene panel sequencing, direct/indirect sequencing, or MLPA testing. Only eleven heterozygous pathogenic variants were found in sixteen unrelated patients (~18%) (16, 18, 20, 21). The results of this study support previous observations of Vaxillaire et al. (21) such as (i) the severity of the clinical presentation of non-European cases (including those of Tunisian ancestry) with early-onset diabetes (similar to F2-IV.12 and F4-IV.8 index cases), and (ii) low mutation rates despite the heterogeneous clinical inclusion criteria [although strict in our study (**Figure 1**)]. In general, this study supports the hypothesis that Tunisian patients have other forms of monogenic diabetes with largely unknown genetic determinants.

## Conclusions

In this study, we report detailed genetic investigation results within suspected MODY patients in Tunisia. Only one patient from the 17 studied index case (5.9%) presented a pathogenic variant; and showed improvement in his long-term monitoring. Moreover, our findings emphasized the importance of exploring the role of variants of uncertain significance in glucose metabolism. Our study proved the efficiency of the whole exome sequencing for MODY diagnosis since more than one major gene might be involved in the emergence of MODY among the Tunisian population.

## Data Availability Statement

The datasets used and/or analyzed in the present study are available from the corresponding author on reasonable request.

## Ethics Statement

The studies involving human participants were reviewed and approved by The Regional Committee of the Protection of Persons, Sfax, Tunisia (CPP SUD N°28/2019). Written informed consent to participate in this study was provided by the participants’ legal guardian/next of kin.

## Author Contributions

All authors listed have made a substantial, direct and intellectual contribution to the work, and approved it for publication.

## Funding

This work was supported by the Ministry of Higher Education and Scientific Research of Tunisia (LR15CBS07 budget), and a scientific research grant from the University of Sharjah (UAE).

## Conflict of Interest

The authors declare that the research was conducted in the absence of any commercial or financial relationships that could be construed as a potential conflict of interest.

## Publisher’s Note

All claims expressed in this article are solely those of the authors and do not necessarily represent those of their affiliated organizations, or those of the publisher, the editors and the reviewers. Any product that may be evaluated in this article, or claim that may be made by its manufacturer, is not guaranteed or endorsed by the publisher.
