# Supplementary material for: Tunisian Maturity-Onset Diabetes of the Young: A Short Review and a New Molecular and Clinical Investigation
Source: Front Endocrinol (Lausanne). 2021 Jul 29;12:684018. doi: 10.3389/fendo.2021.684018 (PMC8358796; doi:10.3389/fendo.2021.684018)
Supplement: Supplementary file 1 [file Table_1.docx]

**Table S1: List of the 133 genes covered by TruSight One Sequencing Panel.**

| **Gene** | **NCBI Reference Sequence** | **HGNC ID** |
| --- | --- | --- |
| *ABCC8* | NM_001287174.2 | 59 |
| *ADRA2A* | NM_000681.4 | 281 |
| *ADCY5* | NM_183357.3 | 236 |
| *AGPAT2* | NM_006412.4 | 325 |
| *AIRE* | NM_000383.4 | 360 |
| *AKT2* | NM_001626.6 | 392 |
| *ALMS1* | NM_001378454.1 | 428 |
| *ANK1* | NM_000037.4 | 492 |
| *ARL6* | NM_001278293.3 | 13210 |
| *ATM* | NM_000051.4 | 795 |
| *BCL11A* | NM_022893.4 | 13221 |
| *BCL2* | NM_000633.3 | 990 |
| *BLK* | NM_001715.3 | 1057 |
| *BSCL2* | NM_001122955.4 | 15832 |
| *CAV1* | NM_001753.5 | 1527 |
| *CDKN2A* | NM_000077.5 | 1787 |
| *CDKN2B* | NM_078487.2 | 1788 |
| *CEL* | NM_001807.6 | 1848 |
| *CEP290* | NM_025114.4 | 29021 |
| *CFTR* | NM_000492.4 | 1884 |
| *CIDEC* | NM_022094.3 | 24229 |
| *CISD2* | NM_001008388.5 | 24212 |
| *DCAF17* | NM_025000.4 | 25784 |
| *DMPK* | NM_001081563.2 | 2933 |
| *EIF2AK3* | NM_004836.7 | 3255 |
| *ERAP2* | NM_001130140.2 | 29499 |
| *FAM58A* | NM_152274.5 | 28434 |
| *FN3K* | NM_022158.4 | 24822 |
| *FOXA1* | NM_004496.5 | 5021 |
| *FOXA2* | NM_021784.5 | 5022 |
| *FOXA3* | NM_004497.3 | 5023 |
| *FOXP3* | NM_014009.4 | 6106 |
| *FTO* | NM_001080432.3 | 24678 |
| *FXN* | NM_000144.5 | 3951 |
| *G6PC2* | NM_021176.3 | 28906 |
| *GATA4* | NM_002052.5 | 4173 |
| *GATA6* | NM_005257.6 | 4174 |
| *GCK* | NM_000162.5 | 4195 |
| *GCKR* | NM_001486.4 | 4196 |
| *GIPR* | NM_000164.4 | 4271 |
| *GLIS3* | NM_001042413.2 | 28510 |
| *GLP1R* | NM_002062.5 | 4324 |
| *GLUD1* | NM_005271.5 | 4335 |
| *GPC6* | NM_005708.5 | 4454 |
| *GRB10* | NM_001001555.3 | 4564 |
| *GRK5* | NM_005308.3 | 4544 |
| *HADH* | NM_001184705.3 | 4799 |
| *HFE* | NM_000410.4 | 4886 |
| *HHEX* | NM_002729.5 | 4901 |
| *HIP1* | NM_005338.7 | 4913 |
| *HK1* | NM_033500.2 | 4922 |
| *HLA-B* | NM_005514.8 | 4932 |
| *HMGA2* | NM_003483.6 | 5009 |
| *HNF-1A* | NM_000545.8 | 11621 |
| *HNF-1B* | NM_000458.4 | 11630 |
| *HNF-4A* | NM_001258355.2 | 5024 |
| *HYMAI* | NR_002768.2 | 5326 |
| *IDE* | NM_004969.4 | 5381 |
| *IER3IP1* | NM_016097.5 | 18550 |
| *IGF1* | NM_001111283.3 | 5464 |
| *IGF2* | NM_000612.6 | 5466 |
| *IKBKAP* | NM_003640.5 | 5959 |
| *IL2RA* | NM_000417.3 | 6008 |
| *INS* | NM_001185098.2 | 6081 |
| *INSR* | NM_000208.4 | 6091 |
| *IRS1* | NM_005544.3 | 6125 |
| *ISL1* | NM_002202.3 | 6132 |
| *KANK1* | NM_001256876.3 | 19309 |
| *KCNJ11* | NM_000525.4 | 6257 |
| *KCNQ1* | NM_000218.3 | 6294 |
| *KL* | NM_004795.4 | 6344 |
| *KLF11* | NM_003597.5 | 11811 |
| *LAMA1* | NM_005559.4 | 6481 |
| *LEP* | NM_000230.3 | 6553 |
| *LEPR* | NM_002303.6 | 6554 |
| *LMNA* | NM_170707.4 | 6636 |
| *LMNB2* | NM_032737.4 | 6638 |
| *LPP* | NM_001167672.3 | 6679 |
| *MADD* | NM_130470.3 | 6766 |
| *MAFB* | NM_005461.5 | 6408 |
| *MAPK8IP1* | NM_005456.4 | 6882 |
| *MC4R* | NM_005912.3 | 6932 |
| *MNX1* | NM_005515.4 | 4979 |
| *MTNR1B* | NM_005959.5 | 7464 |
| *MYT1* | NM_004535.3 | 7622 |
| *NEUROD1* | NM_002500.5 | 7762 |
| *NEUROG3* | NM_020999.4 | 13806 |
| *NOTCH2* | NM_024408.4 | 7882 |
| *PAX4* | NM_006193.2 | 8618 |
| *PAX6* | NM_001258462.3 | 8620 |
| *PCNT* | NM_006031.6 | 16068 |
| *PCSK1* | NM_000439.5 | 8743 |
| *PDX1* | NM_000209.4 | 6107 |
| *PEPD* | NM_000285.4 | 8840 |
| *PIK3CA* | NM_006218.4 | 8975 |
| *PIK3R1* | NM_181523.3 | 8979 |
| *PLAGL1* | NM_001080951.3 | 9046 |
| *PLIN1* | NM_002666.5 | 9076 |
| *POMC* | NM_001035256.3 | 9201 |
| *PON1* | NM_000446.7 | 9204 |
| *PPARG* | NM_015869.5 | 9236 |
| *PPP1R3A* | NM_002711.4 | 9291 |
| *PTEN* | NM_000314.8 | 9588 |
| *PTF1A* | NM_178161.3 | 23734 |
| *PTPRD* | NM_002839.4 | 9668 |
| *RFX6* | NM_173560.4 | 21478 |
| *SGCG* | NM_000231.3 | 10809 |
| *SH2B1* | NM_015503.2 | 30417 |
| *SIRT1* | NM_012238.5 | 14929 |
| *SIX3* | NM_005413.4 | 10889 |
| *SLC16A1* | NM_001166496.2 | 10922 |
| *SLC19A2* | NM_006996.3 | 10938 |
| *SLC29A3* | NM_001174098.2 | 23096 |
| *SLC2A2* | NM_000340.2 | 11006 |
| *SLC30A8* | NM_001172813.2 | 20303 |
| *SOD2* | NM_000636.4 | 11180 |
| *SOX2* | NM_003106.4 | 11195 |
| *SOX9* | NM_000346.4 | 11204 |
| *SPINK1* | NM_003122.5 | 11244 |
| *SPRY2* | NM_005842.4 | 11270 |
| *SRR* | NM_021947.3 | 14398 |
| *SREBF1* | NM_001005291.3 | 11289 |
| *STAT3* | NM_139276.3 | 11364 |
| *TBC1D4* | NM_014832.5 | 19165 |
| *TCF7L2* | NM_001146274.2 | 11641 |
| *THADA* | NM_001083953.2 | 19217 |
| *TRIM32* | NM_012210.4 | 16380 |
| *TTC8* | NM_001288781.1 | 20087 |
| *UCP2* | NM_003355.3 | 12518 |
| *VEGFA* | NM_001025366.3 | 12680 |
| *WFS1* | NM_006005.3 | 12762 |
| *WRN* | NM_000553.6 | 12791 |
| *ZFP57* | NM_001109809.5 | 18791 |

These genes are involved in glucose metabolism, known to cause monogenic diabetes or associated syndromes, and found from genome-wide association data of type 2 diabetes. The molecular function and the Human Phenotype Ontology of each gene has been confirmed according to the VarSome Database (https://varsome.com/). Abbreviation: HGNC, HUGO (Human Genome Organization) Gene Nomenclature Committee (https://www.genenames.org/).
